# The Many Roles of Cell Adhesion Molecules in Hepatic Fibrosis

**DOI:** 10.3390/cells8121503

**Published:** 2019-11-24

**Authors:** Edith Hintermann, Urs Christen

**Affiliations:** Pharmazentrum Frankfurt/ZAFES, Goethe University Hospital Frankfurt, 60590 Frankfurt am Main, Germany; christen@med.uni-frankfurt.de

**Keywords:** cell adhesion, liver fibrosis, selectin, integrin, cadherin, immunoglobulin superfamily, junctional adhesion molecule, non-classical adhesion molecule, antifibrotic therapy

## Abstract

Fibrogenesis is a progressive scarring event resulting from disrupted regular wound healing due to repeated tissue injury and can end in organ failure, like in liver cirrhosis. The protagonists in this process, either liver-resident cells or patrolling leukocytes attracted to the site of tissue damage, interact with each other by soluble factors but also by direct cell–cell contact mediated by cell adhesion molecules. Since cell adhesion molecules also support binding to the extracellular matrix, they represent excellent biosensors, which allow cells to modulate their behavior based on changes in the surrounding microenvironment. In this review, we focus on selectins, cadherins, integrins and members of the immunoglobulin superfamily of adhesion molecules as well as some non-classical cell adhesion molecules in the context of hepatic fibrosis. We describe their liver-specific contributions to leukocyte recruitment, cell differentiation and survival, matrix remodeling or angiogenesis and touch on their suitability as targets in antifibrotic therapies.

## 1. Introduction

The deposition of excess fibrous connective tissue as a result of unrestrained wound healing is a highly conserved process which can afflict any chronically injured organ. Tissue damage is usually detected by resident macrophages, which release proinflammatory mediators to attract other cells of the immune system [1,2,3]. The migration of leukocytes to the site of injury involves generally all main groups of cell adhesion molecules (CAMs) and is a process of extraordinary importance [4,5,6,7]. Therefore, the most thoroughly investigated function of adhesive proteins in the context of inflammation and wound healing is probably their role in vascular exit/extravasation and subsequent tissue immigration. Here we focus on CAMs which have been shown to be relevant to several aspects of chronic liver inflammation and fibrosis. We discuss their function in cell homing to the liver and illustrate their importance for hepatic cells in other activities such as differentiation, survival, contractility or angiogenesis. Then, we address their suitability as clinical markers of hepatic fibrosis and briefly explore antifibrotic therapies which target CAMs in different experimental systems.

## 2. The Liver as Target of Chronic Injury and Fibrosis

The liver has an exceptionally high capacity to respond to injury by tissue repair. However, when an insult persists, repeated damage can induce a wound-healing program which is no longer under control and results in excessive replacement of healthy parenchyma by scar tissue that interferes with normal tissue function. Chronic liver damage can occur due to infection with liver-trophic pathogens (hepatitis B or C virus, worms of the genus *Schistosoma*), drugs/toxins (e.g., acetaminophen), alcoholism, nonalcoholic steatohepatitis (NASH), autoimmune hepatobiliary diseases such as primary sclerosing cholangitis (PSC), primary biliary cholangitis (PBC) or autoimmune hepatitis (AIH) and metabolic disorders such as iron or copper overload (e.g., Wilson’s disease) [8,9,10,11,12]. Fibrogenesis starts with the damage of hepatocytes in parenchymal liver injuries or cholangiocytes in cholestatic diseases that induces the release of inflammatory mediators and the activation of Kupffer cells, which are liver-resident macrophages. In this inflammatory milieu, hepatic stellate cells (HSCs) get activated and differentiate into α-smooth muscle actin (αSMA)-positive myofibroblasts and leukocytes get attracted to the site of injury [11]. They can enter the liver through large blood vessels like the portal vein or the central vein, which both are lined by vascular endothelial cells (ECs) or through the narrow sinusoids consisting of sinusoidal ECs. Due to the small diameter of sinusoids, blood flow in these microvessels is so slow that a deceleration of leukocytes by selectin-mediated tethering and rolling seems not necessary (see classical immigration model, next chapter). Leukocytes rather bind directly via integrin-mediated interaction with endothelial immunoglobulin (Ig) superfamily CAMs or non-classical CAMs [5,13,14]. Once immigrated, leukocytes release more proinflammatory and profibrogenic cytokines, amplifying the inflammatory response and activating additional HSCs, and in biliary diseases also portal fibroblasts, to transdifferentiate into myofibroblasts. Myofibroblasts show a high proliferative, migratory and contractile potential and are the principal producers of fibrotic extracellular matrix (ECM) like fibrillar collagens type I and III, elastin, fibronectin, proteoglycans and others [15,16,17,18,19]. Concomitantly, matrix degradation is reduced, resulting in net matrix accumulation and over time in the disruption of normal tissue architecture, organ dysfunction and eventually organ failure, as well as higher susceptibility to liver cancer. As the given description of the fibrogenic pathway is not detailed enough to account for its complexity, the interested reader is referred to more in-depth reviews [9,11,12,20].

## 3. Cell Adhesion Molecules—General Aspects and their Function in Classical Cell Recruitment

CAMs are generally divided into four major groups: Selectins, integrins, cadherins and members of the Ig superfamily of CAMs, IgCAMs [4,21,22,23,24,25,26,27,28]. In addition to these conventional CAMs, non-classical CAMs like vascular adhesion protein 1 (VAP-1), mucosal addressin cell adhesion molecule 1 (MAdCAM-1) or stabilins are also of importance in cell adhesion [29,30,31,32,33]. CAMs enable cells to interact with other cells and/or with the ECM, influencing a wide variety of fundamental processes like tissue organization and remodeling, inflammation and repair or cell survival and malignant transformation. These mostly transmembrane glycoproteins allow resident cells like endothelial and epithelial cells to form stable contacts with their immediate neighbors and the substrate they reside on and patrolling cells such as lymphocytes or neutrophils to temporarily attach to cellular and matrix surfaces during their journey. Intracellularly, CAMs associate with scaffolding proteins and signaling molecules, which upon binding transfer signals from the outside to the cell interior (outside-in signaling) and thereby modulate cell behavior in response to the microenvironment [23,24,25,28]. In addition to adhesion-induced signaling, CAMs can mediate adhesion-independent signaling due to their interaction with growth factor receptors, transcription factors or other signaling proteins [23,24,25,28]. Activation of cells by soluble molecules like chemokines can lead to intracellular events that induce conformational changes in extracellular domains of the adhesive proteins, e.g., integrins, regulating the affinity for extracellular ligands (inside-out signaling) [24,26,27]. Under inflammatory conditions, activation and recruitment of immune cells from the vascular lumen to the site of insult are crucial steps in the disease process and involve leukocyte adhesion to and transmigration of the endothelium, migration into and within tissues, binding to target cells and execution of cytotoxic reactions. Endothelial and epithelial cells support these processes by upregulating CAM expression and secreting inflammatory mediators [4,7,23,34]. Thus, in the classical immigration model, leukocytes following chemokine gradients in the blood first tether to and then roll along vascular ECs with the help of selectins. This step slows leukocytes down and allows them to interact with endothelium-bound chemokines, which activate leukocyte integrins. These integrins increase affinity and avidity for their endothelial adhesion receptors, which frequently belong to the IgCAM family, resulting in firm adhesion. After recruitment, leukocytes either squeeze through endothelial junctions (paracellular route), a process promoted by cadherins and IgCAMs, or migrate through the body of ECs (transcellular route), again supported by IgCAMs [4,23,34]. Then, they traverse the perivascular basement membrane and finally enter the inflamed tissue.

## 4. Adhesion Molecules in Cell–ECM Interaction

The ECM is a 3D network composed of proteins, non-protein components and bound soluble factors like cytokines and growth factors, which delivers ECM-derived signals via integrins and cytokine/growth factor receptors to cells on-site. The tissue-specific ECM composition defines the homeostatic cell phenotype and communicates dynamic changes in the microenvironment, as induced during disease [35]. For example, naïve HSCs are located in the sub-endothelial space of Dissé where they assemble a low-density ECM, which maintains a state of quiescence in HSCs and sinusoidal ECs and is crucial for epithelial polarity in hepatocytes [36,37]. Upon activation, myofibroblastic HSCs progressively transform the surrounding ECM into a high-density matrix with increased stiffness. To adapt cell–matrix interactions to these changes in ECM structural and signaling properties, liver cells can modulate their repertoire of ECM receptors. These adjustments together with HSC-derived vascular endothelial growth factor (VEGF) and sinusoidal platelet-derived growth factor (PDGF) and transforming growth factor β (TGFβ) stimulate cell migration and sinusoid formation and contribute to fibrosis-associated angiogenesis in the liver [36,38]. Furthermore, fibrotic ECM impairs hepatocyte polarity and function [37] and can enhance cholangiocyte proliferation in cholestatic liver disease [39,40].

A general key regulator of tissue fibrosis is soluble TGFβ, which exists in three isoforms with overlapping functions. Expression levels of all isoforms are increased in cirrhotic human livers [41], indicating its involvement in disease progression. Each isoform is synthesized as precursor protein that gets proteolytically cleaved, resulting in a C-terminal fragment which assembles as a disulfide-linked homodimer. This mature cytokine is encased within a larger amino-terminal fragment called latency-associated peptide (LAP), forming the “small latent complex”. LAP prevents the C-terminal mature cytokine from binding to its receptor. The small latent complex is linked to the so-called “latent TGFβ binding protein”, which is tethered to the ECM and thereby stores TGFβ extracellularly in an inactive form. TGFβ1 and TGFβ3 can be released via conformational changes in the latency cage induced by arginine-glycine-aspartate (RGD)-binding integrins, specifically members of the αV-integrins like αVβ1 or αVβ6 [42,43]. Like this, mechanical forces, which are generated by integrin-mediated regulation of the actin cytoskeleton, e.g., during cellular contraction, can generate active TGFβ [44,45] that in return may increase the expression of integrins. Furthermore, TGFβ release is facilitated by stiffening of the surrounding ECM [18], suggesting that fibrotic ECM, which contains a high portion of fibrillar collagens, increases the bioavailability of latent TGFβ. TGFβ, in turn, activates HSC differentiation to a myofibroblast phenotype, which subsequently synthesizes more fibrotic ECM that is then fed into the self-perpetuating cycle of tissue fibrosis. Triggering TGFβ activation is further possible via αVβ8-mediated co-localization of matrix metalloproteinases with the large latent TGFβ complex, resulting in proteolytic release of active TGFβ [42]. Due to the absence of an RGD sequence in TGFβ2, this isoform is released integrin-independently by proteases or thrombospondin-1 [46]. In addition to its function as major profibrotic cytokine, TGFβ acts also as potent repressor of epithelial, e.g., hepatocyte, proliferation [47,48] and as co-activator of the proinflammatory Th17 lymphocyte subset [49,50]. Thus, integrins which trigger TGFβ activation can boost chronic liver injury by blocking hepatocyte regeneration, by promoting differentiation of proinflammatory Th17 lymphocytes and by increasing myofibroblast numbers and fibrotic ECM.

## 5. Adhesion Molecules in Cell–Cell Interaction

Cell–cell interactions on the one hand can be long-lasting when formed between cholangiocytes integrated in a functional bile duct or ECs forming a mature blood vessel, but on the other hand they can be a brief handshake when leukocytes adhere to ECs during their voyage through the vasculature. With regards to CAMs and their functions in hepatic inflammatory diseases, current investigations focus strongly on their central role in sinusoidal endothelium-mediated leukocyte recruitment. The increase in leukocyte immigration into the inflamed liver is not only dependent on specific cell attraction by released chemokines but is further boosted by an upregulation of CAM levels, which allow faster cellular turnover at the sites of hepatic entry. As discussed later, the expression of many CAMs is stimulated in the inflammatory milieu by lipid-derived mediators, cytokines or chemokines such as interferon γ (IFNγ), tumor necrosis factor α (TNFα) or interleukin 1β (IL-1β).

Another important fact is that chronic inflammation and fibrosis are accompanied by distortion of the liver vasculature, resulting in dysregulation of normal blood flow and dynamic rise in portal pressure. The normal hepatic microvasculature is composed of fully differentiated sinusoidal ECs, which contain transmembrane pores called fenestrations, forming a discontinuous barrier with strong transvascular exchange [51]. These ECs attract HSCs as pericytes, which embrace the sinusoids with their long cytoplasmic processes, thus stabilizing vessel walls [51,52]. Additionally, HSCs secrete VEGF to support sinusoidal EC differentiation. In return, differentiated sinusoidal ECs prevent HSC activation [53]. During fibrogenesis, sinusoidal ECs de-differentiate and lose their fenestrations, a process termed capillarization. Further, activated HSCs become more numerous and increase their contractile force in response to TGFβ, resulting in extensive coverage of the sinusoid wall by pericytes and a reduction in sinusoid diameter. During these processes, homotypic and heterotypic cell–cell interactions between HSCs and ECs intensify due to increased CAM levels [54]. In addition to sinusoidal capillarization and pericytic constriction, fibrotic ECM compresses portal and central veins, leading to increased resistance to blood flow and hence reduced oxygen delivery. This results in hypoxia and the release of hypoxia-inducible proangiogenic factors, which stimulate neovascularization [38]. New vessels promote inflammation and fibrosis as they offer more dock sites to migrating leukocytes. Hence, angiogenesis and fibrosis develop in parallel and blocking angiogenesis has reduced hepatic fibrosis in several experimental models [38,55,56]. However, as discussed in the chapter on integrins, the relationship between these two processes has turned out to be more complicated than initially thought.

In the context of CAMs and liver fibrosis, the concept of epithelial to mesenchymal transition (EMT) needs to be briefly mentioned, as loss of intercellular adhesive structures, among other key epithelial characteristics, is needed to promote rapid mobilization of large numbers of fibrogenic cells. In fact, in many cases of acute and chronic hepatic injury, CAMs on hepatocytes get downregulated and the formation of cell junctions is inhibited [57]. However, since some reports support and others deny the ability of hepatocytes and/or cholangiocytes to complete EMT, the topic is still a matter of debate and the reader is referred to some in-depth reviews [58,59,60].

In the following paragraphs we will discuss the contribution of each of the four CAM groups to hepatic fibrosis in rodents and men.

## 6. Selectins

The selectin family of adhesion molecules comprises three members, i.e., L-, E-, and P-selectin (Table 1), which are mucin-like calcium-dependent CAMs expressed on ECs, leukocytes and platelets and contain a lectin domain and several complement binding repeats in their extracellular portion [61,62,63]. Selectins are known to act as primary capture receptors allowing leukocytes to tether and roll along the blood vessel wall before they firmly bind to and then migrate through the EC layer, using integrins and IgCAMs. In fact, the interaction between selectins and their ligands primes integrin activation through inside-out signaling, which allows cell rolling, followed by additional chemokine receptor signaling, triggering full activation of integrins. However, studies on the role of selectins in liver fibrosis are scarce and have generated contradictory results in rodents, which either affirm or deny a critical function of selectins during liver infiltration of leukocytes [64,65,66,67,68]. One critical point that determines the outcome of these experiments is the site of analysis. Intravital microscopy studies have demonstrated that about 80% of leukocytes adhere within the sinusoids and just a minority in postsinusoidal venules [68]. However, only in the latter, selectin-dependent rolling and adhesion have been observed [66,68]. Importantly, the discrepancy in these studies may also be due to the different treatments applied. Liver damage induced by a 4-hour LPS challenge was similar in wild-type and E-selectin/P-selectin-deficient mice [68], whereas 9 hours after binge feeding of chronically ethanol treated mice, liver injury and inflammation were lower in E-selectin-deficient mice than in wild-type animals [64]. Also, some blocking experiments demonstrated a proinflammatory effect of selectins as inhibition of selectin function by fucoidan prevented murine liver damage induced by a 24-h challenge with TNFα and galactosamine [66] and antibody blockade of P-selectin glycoprotein ligand-1 (PSGL-1) reduced leukocyte rolling and adhesion 12 hours after bile duct ligation [65]. Further, the L-selectin ligand MECA-79 was detected after ischemia reperfusion injury in steatotic but not normal murine livers [67]. Since in all these experiments the possible effect of selectins was analyzed within a few hours after the liver was challenged, results are relevant to acute inflammation rather than chronic inflammation and fibrosis. More significant in this regard is the following study by Wynn et al. In P-selectin-deficient mice, hepatic fibrosis induced by the parasite *Schistosoma mansoni* and analyzed 16 weeks after infection was dramatically increased compared to livers of wild-type mice and correlated with a higher frequency of liver-infiltrating IL-13- and IFNγ-producing lymphocytes as well as a reduction in decoy IL-13 receptor expression. These results suggest that in mice P-selectin may protect from liver fibrosis by suppressing an IFNγ response and supporting decoy IL-13 receptor synthesis [69]. Analyses of human biopsies have shown that selectins are absent on sinusoidal and vascular ECs in the healthy liver and levels of E- and P-selectin increase only on vascular but not sinusoidal ECs during inflammation (Table 1). Furthermore, expression of E-selectin ligands was low independent of the cause of inflammation [5,70]. These findings suggest that selectins play a minor role in hepatic leukocyte recruitment in men, making it necessary for liver-infiltrating cells to use other adhesion molecules as liver homing receptors [5,71].

## 7. Integrins

Integrins are heterodimeric glycoproteins consisting of an α- and a β-chain which associate with numerous intracellular adaptor- and signaling molecules in specialized structures called focal contacts or focal adhesions, linking them to the actin cytoskeleton. In mammals, 18 α-chains can assort non-covalently with 8 β-chains to form at least 24 distinct integrins [27]. These cell surface receptors integrate cells with their microenvironment by either binding to ECM ligands like fibronectin, laminins or collagens, or by interacting with non-ECM proteins like counter-receptors on adjacent cells during leukocyte transmigration of tissue or tissue damage by leukocytes (Table 1). Additional non-ECM ligands are, e.g., growth factors, hormones, venoms or viral and bacterial proteins [72]. Observations that ECM acts as reservoir for growth factors/cytokines and that integrins are involved in growth factor receptor signaling point out why integrin functions go way beyond anchoring cells to their substrate or their neighboring cells [73]. Therefore, integrin repertoire and integrin expression levels correlate closely with the functional capacity of an immigrated cell. For example, active neutrophils show higher αMβ2 levels than inactive ones and neutrophil cytotoxic activity can be blocked with a monoclonal antibody to αM [74] or genetic ablation of β2 [75], preventing neutrophils from binding to hepatocytes and harming them. Similarly, in a murine malaria model, only those cytotoxic CD8^+^ T cell clones which expressed high levels of α4β1 (VLA-4) showed a strong anti-parasite effect, since solely those clones were able to interact with parasitized hepatocytes to kill them [76]. Clinical studies have shown that the levels of certain integrin chains are upregulated in patients with chronic liver diseases and correlate with the stage of fibrosis [77,78]. Below we will summarize some of the most important aspects of integrin biology in connection with hepatic fibrosis.

### 7.1. β1-Integrins

Integrins called very late activation antigens (VLA-1 to VLA-6) are a subfamily of the β1-integrins. The essentially leukocyte-specific α4β1 (VLA-4) is expressed mainly on T cells and monocytes and to a lesser extent on neutrophils and plays a major role in cell recruitment to infected or inflamed tissue through binding to IgCAMs, whereas binding to ECM is less important [27,79,80]. The interaction between α4β1 and VCAM-1 activates outside-in signaling that promotes T cell survival and delays apoptosis [81], thus extending immune reactions in the injured tissue, e.g., the liver. All other VLA integrins are present in the healthy human liver on vascular and sinusoidal ECs, hepatocytes and cholangiocytes with varied cell specificities and they mediate binding to collagens, laminins and fibronectin (Table 1) [27,82]. Studies by Volpes et al. have shown that chronic inflammation due to HBV or HCV infection, alcohol abuse or cholestatic diseases induced strongest VLA level changes in hepatocytes as they upregulated α1β1 and α5β1 expression and de novo synthesized bile duct-specific α2β1, α3β1 and α6β1. This implies that hepatocytes experience a phenotypic switch to cholangiocytes in cholestatic diseases. Sinusoidal ECs increased the α5β1 level and de novo synthesized α4β1, whereas vascular ECs (express all VLA integrins except α4β1) and cholangiocytes showed no significant changes [82]. The observed gain in VLA integrins reflects the increase in laminins, collagens and fibronectin typically found in fibrotic tissue and may represent an adaptive response to prevent epithelial loss due to on-going tissue injury. Integrins α1β1, α2β1 and α5β1, together with the more recently identified α11β1, are expressed on HSCs and allow them to sense differences in matrix composition [83,84]. A higher content of fibrillar collagens results in increased substrate stiffness, which boosts HSC activation, perpetuating fibrogenesis. Hence, these integrins play a minor role in tissue homeostasis but are instrumental during dynamic connective tissue remodeling, as occurring during fibrogenesis [84]. In fact, genetic inactivation of β1 [85] and depletion of α11 by a lentiviral sh-RNA approach [86] reduced HSC differentiation, migration, contractility and ECM production in vitro and allowed the identification of signaling pathways associated with α11β1 profibrotic functions. Interestingly, pharmacological inhibition of either pathway reduced collagen I expression in carbon tetrachloride (CCl_4_) treated and bile duct ligated mice and in human liver slices ex vivo [85,86], suggesting that α11β1 plays a promoting role in hepatic fibrogenesis. Other collagen-binding β1-integrins, such as α1β1 or α2β1 are less well studied in the liver [84,87,88].

### 7.2. β2-Integrins

Similar to α4β1, the β2-integrins, also called leukocyte integrins, are indispensable for tissue immigration [27,79,89,90]. Unlike other integrins, they have only few ECM ligands since their main function is to enable adhesion to other cells through binding to members of the IgCAM superfamily, e.g., ICAMs, VCAMs or JAMs. Integrin αLβ2, also called lymphocyte function-associated antigen-1 (LFA-1) is present at high levels on all leukocytes. Integrins αMβ2 (macrophage-1 antigen, Mac-1), αXβ2 (p150,95) and αDβ2 are also widely expressed on leukocytes [27,90]. The β2 expression level is increased on leukocytes of patients with liver cirrhosis [91]. The role of αLβ2/ICAM-1 interaction in T cell activation will not be discussed here but is the topic of several reviews [92,93,94,95,96].

### 7.3. αV-Integrins

An integrin subfamily which has attracted much attention in the fibrosis field in recent years are the αV-integrins, αVβ1, αVβ3, αVβ5, αVβ6 and αVβ8, since they can release active TGFβ. Apart from αVβ6, all αV-integrins are present on HSCs (Table 1), with αVβ1 predominantly activating TGFβ [44,97], hence promoting HSC differentiation and hepatic fibrosis. In fact, a small molecule αVβ1 inhibitor, which was able to down-regulate TGFβ-induced signaling, was effective in reducing CCl_4_-induced hepatic fibrosis in mice [98]. These data support the elegant studies by Henderson et al. which demonstrated that HSC-specific targeted deletion of αV, but not global lack of β3, β5 or β6, or HSC-specific deletion of β8, protects from CCl_4_-induced liver fibrosis [97] due to a significant drop in the level of active TGFβ. Proliferation was not impaired in αV-null HSCs, although earlier studies by Zhou et al. had shown that a neutralizing anti-β3 antibody and small inhibitory RNA to silence αV induced HSC apoptosis [99]. Another interesting HSC activation mechanism which is dependent on αV-integrins was described by Muhanna et al. The authors co-cultured HSCs with T cells or NK cells, since in biopsies of HBV and HCV patients with advanced fibrosis HSCs showed direct interaction with CD4^+^ and CD8^+^ T cells in vivo. They observed that T cells but not NK cells triggered increased αSMA expression in HSCs. Furthermore, T cells from patients but not healthy controls were phagocytosed by HSCs. Phagocytosis and HSC activation were blocked with antibodies to the IgCAM member ICAM-1 and to αV-integrin, suggesting that ICAM-mediated HSC/T cell interaction/phagocytosis and αV-integrin ligation stimulated HSC differentiation [100]. Whether this effect is due to CAM outside-in signaling and/or TGFβ-dependent was not yet analyzed in detail. Of note, phagocytosis of hepatocyte apoptotic bodies by HSCs stimulated HSCs as well and was dependent on TGFβ1 release [101,102]. Integrins αVβ3 and αVβ5 are present on ECs, also in the liver (Table 1), promoting migration, proliferation and blood vessel formation. They are the main targets of angiogenesis inhibitors used for the treatment of cancer [56]. Due to the previously mentioned positive correlation between angiogenesis and liver fibrosis, αVβ3 and αVβ5 represent therapeutic targets whose inhibition could potentially reduce both fibrogenesis and neovascularization. In fact, blockade of αVβ3/αVβ5 with a small molecule inhibitor reduced angiogenesis but at the same time increased fibrosis induced by bile duct ligation or thioacetamide in rats [103]. This was unexpected, since inhibition of αVβ3 blocked HSC proliferation at least in vitro [99,103] and antiangiogenic treatments reduced CCl_4_-induced fibrosis in mice [104,105]. Because αVβ3 blockade induced mild hypoxia, macrophage activation could be the reason for stronger hepatic fibrosis when αVβ3/αVβ5 were inhibited in these animal models [103]. An aspect which complicates angiogenesis blockade via αVβ3 and αVβ5 further is their possible interplay with the VEGF/VEGF receptor-2 system. As published by Reynolds et al., β3-deficient and β3/β5-deficient mice responded with increased angiogenesis and showed higher levels of endothelial VEGF receptor-2 compared to wild-type mice in a tumor model [106]. The authors discuss the possibility that αVβ3 and αVβ5 usually regulate the VEGF/VEGF receptor-2 system, which could be dysregulated in the absence of integrin control. Whether the same crosstalk is active in liver ECs remains to be tested. Integrin αVβ6 is generally found on injured or inflamed epithelia, mediating binding to fibronectin and tenascin-C (Table 1) and supporting cell proliferation during wound healing [44,107]. In the liver, integrin αVβ6 is expressed on activated cholangiocytes, transitional hepatocytes [108] and oval cells during biliary and portal fibrosis [108,109,110]. Blockade of αVβ6 by a non-peptide antagonist lowered biliary fibrosis triggered by bile duct ligation in rats. The observed inhibitory effect was dependent on reduced release of active TGFβ [108,109,110]. Integrin αVβ8 is present on hepatocytes and has a control function during liver regeneration after injury, as it blocks hepatocyte proliferation through the release of TGFβ. Similarly, TGFβ may prevent repair mechanisms in fibrotic tissue by inhibiting hepatocyte growth [111]. In conclusion, integrins can be upregulated by proinflammatory mediators and subsequently maintain inflammation by recruiting leukocytes to the site of injury. Some αV-integrins can directly expedite fibrosis by activating HSC and portal fibroblast differentiation through the release of TGFβ and by promoting angiogenesis.

## 8. Cadherins

Cadherins are a family of calcium-dependent transmembrane proteins, which are subdivided into classical/major cadherins, protocadherins and cadherin-related family members [112,113,114,115,116]. They are the core components of the intercellular adhesive structures called desmosomes and adherens junctions, which provide solid tissues with mechanical and functional integrity as they mediate homotypic cell–cell adhesion and form barriers either between organ compartments or between the body and the external environment. Extracellularly, cadherins dimerize homophilically both in cis (on same cell) and in trans (between neighboring cells) forming a zipper-like structure. Intracellularly, these adhesion molecules are anchored to cytoskeletal filaments and scaffolding complexes, which contain signaling proteins that regulate cell adhesion, migration and polarity. During leukocyte transmigration, cadherins and associated proteins transiently disappear from the cell membrane but rapidly reconstitute junctional complexes after leukocytes have passed [4].

Desmosomes are submembranous plaques in epithelial but not endothelial cells, which offer strong resistance to mechanical and shear stress [117]. They contain the two cadherins desmoglein and desmocollin (Table 2), which allow tight cell–cell interaction via homophilic or heterophilic binding and connect with intermediate filaments via a series of cytoplasmic proteins like the armadillo family proteins plakoglobin (γ-catenin) and plakophilins, and with plakins [24,57,117]. They not only enable association between cadherins and keratin intermediate filaments but link cadherins with important signaling pathways like the canonical Wnt pathway [117]. The exact localization of desmoglein and desmocollin in hepatic epithelial cells has not been determined yet [57] and mutations in desmosomal proteins affect mostly the heart and the skin. However, Zhou et al. have shown that in the absence of plakoglobin, bile duct ligation resulted in a more severe disease outcome with enhanced liver fibrosis [118]. This result suggests that intact desmosomes are mandatory to prevent tissue injury due to increased backpressure and shear stress resulting from ligation-induced bile accumulation in intrahepatic bile ducts. In fact, desmosomes are more numerous in patients with extrahepatic cholestasis than in healthy controls [119], demonstrating a natural compensatory mechanism.

Cadherins are further present in adherens junctions which constitute either a continuous circumferential belt (zonula adherens) around polarized cells like epithelial and endothelial cells or form lateral spot-like adhesions (puncta adhaerentia) like in fibroblasts [22,24]. Similar to desmosomal cadherins, E-cadherin in epithelial and VE-cadherin in endothelial tissues are transmembrane proteins, which need to tether with members of the catenin family (α-catenin, β-catenin, p120-catenin and plakoglobin) in order to link with the actin cytoskeleton or microtubules and associated signaling pathways, such as the canonical Wnt pathway [24]. The role of Wnt/β-catenin signaling in liver homeostasis and injury is the subject of excellent reviews by S.P. Monga [120,121]. In mice, E-cadherin expression is observed in cholangiocytes and periportal hepatocytes, whereas perivenous hepatocytes are E-cadherin-negative (Table 2). Interestingly, the absence of E-cadherin or p120-catenin had no negative effect on murine hepatocyte differentiation and homeostasis, but impaired cholangiocytes, which showed spontaneously reduced differentiation and increased proliferation, leading to faulty bile duct formation and strong periductal fibrosis, resembling PSC in men [122,123]. In fact, in four out of seven PSC biopsies, E-cadherin expression was defective on cholangiocytes, but not hepatocytes [122]. These findings point out that an intact intrahepatic biliary network with normal bile secretion depends on functional E-cadherin and p120-catenin. Another interesting observation is that E-cadherin can bind to αEβ7-integrin [124]. This interaction could by mistake mediate the positioning of αEβ7-positive mucosal T cells near cholangiocytes and hepatocytes inducing a proinflammatory environment similar to MAdCAM-1 (see section on non-classical CAMs). This effect might be further boosted by TGFβ as it upregulates mRNA levels of αE and β7 [124]. E-cadherin is also expressed in quiescent HSCs but gets lost when HSCs differentiate into myofibroblasts, which then instead express N-cadherin (Table 2) [125,126]. Interestingly, HSC activation is blocked when E-cadherin is overexpressed, suggesting an antifibrotic potential of E-cadherin. In fact, E-cadherin interferes with TGFβ1 signaling by recruiting RhoA to the p120-catenin binding site, thus preventing RhoA-dependent Smad3 signaling, resulting in reduced expression of TGFβ1 and its downstream genes in HSCs [127]. In addition to myofibroblasts, N-cadherin is also expressed by hepatocytes, but not cholangiocytes or sinusoidal ECs [128,129], suggesting that myofibroblast-sinusoidal interaction is not mediated by N-cadherin, as it is in the case of other vascular beds [25]. Rather, N-cadherin mediates homotypic myofibroblast interaction and supports myofibroblast survival, as N-cadherin blockade by antibodies or cleavage by matrix metalloproteases induced myofibroblast apoptosis [125,126,130]. This raises the question whether myofibroblast-specific N-cadherin blockade could reduce liver fibrosis.

A classical cadherin with a prominent role in fibrogenesis is cadherin-11, which is upregulated on activated macrophages and on myofibroblasts in fibrotic lung and skin tissue [131,132]. Work by Lodyga et al. demonstrated that cadherin-11 mediates the association between myofibroblasts and macrophages, resulting in an increase in local TGFβ concentration as activated macrophages provide latent TGFβ and fibroblasts can activate this cytokine, concurrently responding with increased differentiation [133]. Interestingly, tissues like bladder or skin in cadherin-11-deficient mice showed a considerably reduced mechanical strength, accompanied by a lowered collagen and elastin content compared to wild-type tissues [134]. These results can be explained by the observation that homophilic cadherin-11 binding can trigger increased collagen and elastin secretion in mouse and human dermal fibroblasts, an effect which was TGFβ dependent [134]. These studies identified cell–cell adhesion as regulator of TGFβ-induced ECM production. In the murine liver, expression of cadherin-11 is low in the healthy organ but increased upon CCl_4_ treatment on hepatocytes, HSCs and macrophages (Table 2) [135]. Also, in human livers cadherin-11 levels correlated with the fibrosis stage [136]. Genetic deletion of cadherin-11 protected mice from CCl_4_-induced liver fibrosis and resulted in reduced expression of collagen I and αSMA as judged by immunohistochemical analysis and quantitative PCR [135]. It would be interesting to further determine elastin levels and measure liver elasticity in this animal model.

## 9. Immunoglobulin Superfamily of Adhesion Molecules

The calcium-independent immunoglobulin superfamily of CAMs (IgCAMs) represents one of the largest and most diverse protein families. As implied by the name, all IgCAM members contain at least one immunoglobulin or immunoglobulin-like domain in their extracellular portion. Most IgCAMs are transmembrane proteins but some are linked to the cell surface by a glycophosphatidyl inositol anchor. The cytoplasmic tail is linked to components of the cytoskeleton and to various signaling proteins [21,23]. IgCAMs are not associated with specific adhesive structures but instead are distributed along intercellular boundaries. Well-known members are major histocompatibility complex (MHC) class I and II molecules, proteins of the T cell receptor (TCR) complex, intercellular adhesion molecules (ICAMs), vascular cell adhesion molecules (VCAMs), platelet-endothelial cell adhesion molecule 1 (PECAM-1), neural cell adhesion molecule (NCAM), junctional adhesion molecules (JAMs), nectins, and nectin-like proteins. During tissue injury and inflammation, MHC molecules and TCR are mainly involved in T cell activation, whereas the other IgCAM members, which we will discuss here (Table 2), mediate primarily cell–cell adhesion important for cell recruitment, differentiation or angiogenesis [21].

### 9.1. ICAMs

Members of the intercellular adhesion molecule (ICAM) subfamily are located on endothelial and epithelial cells and are further expressed by fibroblasts, keratinocytes and leukocytes [26]. They are the main receptors for β2-integrins like αLβ2 allowing firm leukocytes adhesion, as well as transcellular or paracellular migration [4,89]. ICAM expression is upregulated in various inflammatory, autoimmune or allergic diseases by inflammatory cytokines such as IFNγ, IL-1β or TNFα and by reactive oxygen species [137,138]. In the healthy human liver, ICAM-1 is constitutively expressed by ECs of the entire vasculature (Table 2). Liver injury triggers increased endothelial expression and de novo synthesis by hepatocytes and cholangiocytes, though the latter happens specifically in biliary diseases such as PSC and PBC [139,140,141,142]. Cytokine-induced upregulation of ICAMs is the consequence rather than the cause of chronic inflammation and promotes liver-specific recruitment of leukocytes to the site of hepatic injury [143], as discussed before. Interestingly, bile duct ligation in rats induced ICAM-1 expression on activated HSCs [144] and HSC/T cell interaction mediated by ICAM-1 stimulated HSC differentiation [100], as mentioned in the chapter on integrins. Thus, besides facilitating leukocyte immigration into the inflamed liver, ICAM-1 may also support immunomodulatory functions of activated HSCs by enabling direct interaction between HSCs and infiltrating T cells [145].

### 9.2. VCAMs

Vascular cell adhesion molecule (VCAM) proteins are present mainly on ECs but also on epithelial cells and are the main receptors for β1-integrins like α4β1. Similar to ICAMs they support firm leukocyte adhesion and transcellular or paracellular migration [26]. Hepatic VCAM-1 was only weakly expressed on human portal ECs [13] but like ICAM-1, VCAM-1 expression was induced or upregulated by inflammatory cytokines and therefore detected at higher levels on vascular and sinusoidal ECs in inflammatory liver diseases. In contrast to ICAM-1, VCAM-1 was usually only rarely observed on bile ducts of PSC or PBC patients [13]. However, in a more recent report, Afford et al. have shown that prominent cholangiocyte expression of VCAM-1 was not restricted to biliary diseases but was also present in alcoholic liver disease and chronic hepatitis C [81]. Interestingly, in an antigen-driven mouse model of biliary injury, VCAM-1-mediated adhesion of α4β1-positive hepatic T cells to cholangiocytes reduced apoptosis, thus promoting T cell survival and continuance of hepatic inflammation. Since IFNγ and TNFα can trigger VCAM-1 expression in cholangiocytes in vitro, T cells themselves may prolong their own survival by secreting these chemokines to stimulate cholangiocytes [81].

### 9.3. PECAM-1

Platelet/endothelial cell adhesion molecule-1 (PECAM-1, CD31) is composed of six Ig-like domains, one transmembrane domain and a cytoplasmic tail with ITIM domains, which can participate in outside-in and inside-out signaling [146]. PECAM-1 is expressed by ECs at intercellular junctions outside of adherens and at tight junctions, on platelets, on all leukocytes like neutrophils, monocytes, NKs and selected T cell subsets, mediating either homophilic interaction or heterophilic binding to integrin αVβ3 on ECs or CD177 on neutrophils [146,147,148,149]. PECAM-1 on leukocytes is involved in chemokine-mediated directional migration to the site of inflammation. Endothelial PECAM-1 is a major mechanosensor, a modulator of vascular permeability by maintaining EC barrier function and a regulator of leukocyte trafficking. As such, PECAM-1 mediates leukocyte paracellular and transcellular migration through the endothelial layer and supports cell migration through basement membranes by translocating integrin α6β1 to the neutrophil cell surface [146,147,149]. Furthermore, PECAM-1 homophilic interaction triggers signaling events which lead to the activation of β1- and β2-integrins on leukocytes. Thus, by facilitating leukocyte trafficking, PECAM-1 plays an important proinflammatory role. In addition, PECAM-1 performs anti-inflammatory functions, including the maintenance of vascular barrier integrity, inhibition of proinflammatory cytokine production, and suppression of leukocyte activation. Furthermore, it plays a key role in regulating T cell survival and effector function [148]. All these aspects of PECAM-1 function have been analyzed in a multitude of animal models, which have revealed that in most mouse strains the pro- and anti-inflammatory properties of PECAM-1 offset each other, whereas in C57BL/6 mice the anti-inflammatory effect dominates [146,147,149]. Hepatic expression of PECAM-1 is controversially discussed. Chosay et al. detected PECAM-1 on vascular, but not sinusoidal, ECs in the healthy mouse liver and observed no change in expression due to liver injury by endotoxemia [150]. Straub et al. demonstrated PECAM-1 expression on naïve mouse sinusoidal ECs which was upregulated on capillarized ECs isolated from livers chronically exposed to the hepatotoxin arsenic [151]. In our hands, naïve murine sinusoidal ECs were also PECAM-1-positive, and its expression was increased during chronic CCl_4_ treatment [152]. Neubauer et al. reported that in livers of naïve rats sinusoidal ECs were PECAM-1-positive, whereas acute treatment with CCl_4_ decreased PECAM-1 expression. Interestingly, in vitro PECAM-1 levels were found to be decreased on vascular ECs or neutrophils by TNFα and IFNγ, whereas TGFβ treatment upregulated PECAM-1 expression [153]. This result may explain why PECAM-1 expression was downregulated after acute CCl_4_ treatment but upregulated after chronic CCl_4_ administration. In healthy human livers, sinusoidal ECs were PECAM-1-negative but in cirrhotic livers, these cells did express PECAM-1 [154]. The role of PECAM-1 in liver fibrogenesis has so far only rarely been investigated. PECAM-1-deficient mice showed stronger liver injury and fibrosis in a diet-induced steatohepatitis model [155], whereas fibrosis was less severe after chronic thioacetamide treatment [156]. These contradictory results further illustrate the complexity of PECAM-1 as a protein with adhesive and signaling functions in the immune and the vascular system and call for more studies to identify its precise roles in chronic liver inflammation and fibrosis.

### 9.4. NCAM

Neural cell adhesion molecule (NCAM) was shown to mediate cell–cell adhesion but also cell/ECM interaction by binding to NCAM or N-cadherin as well as proteoglycans or agrin, respectively [157]. In the normal human liver, NCAM expression is restricted to nerve fibers in the portal tract. However, in cholestatic diseases, also cholangiocytes and myofibroblasts were found to be NCAM-positive [158]. In rodents, bile duct ligation and CCl_4_ treatment triggered NCAM expression in myofibroblasts [159] and HSCs isolated from NCAM-deficient mice showed impaired differentiation compared to cells from wild-type mice, explaining the weaker bile duct ligation-induced fibrogenesis observed when NCAM was knocked-out [157]. Further studies are needed to determine the role of NCAM in hepatobiliary diseases.

### 9.5. JAMs

The junctional adhesion molecule (JAM) subfamily consists of three classical JAMs (JAM-A, B, and C) and four related proteins (JAM-4, JAM-L, CAR and ESAM). In this review, we will focus on classical JAMs only. All members are composed of two Ig-like domains, one transmembrane domain and a cytoplasmic tail with a PDZ domain binding motif, which can participate in intracellular signal transduction [4,160,161]. The extracellular domains form in trans homophilic (all JAMs) and heterophilic (JAM-B with JAM-C) interactions but bind also to integrins: JAM-A interacts with αLβ2 and αVβ3, JAM-B binds α4β1 and JAM-C associates with integrins αMβ2, αXβ2 and αVβ3 (Table 2) [160]. Furthermore, JAMs can influence integrin localization and activity either by direct association and/or by intracellular signaling. For example, JAM-C can interact in cis with αVβ3 thereby inhibiting integrin activity by blocking the small GTPase Rap1 [162]. In contrast, JAM-A does not interact with β1-integrin but decreases integrin activity also through Rap1 inhibition [161]. JAM-A and JAM-C are present on endothelial and epithelial cells, on platelets and on leukocytes in humans. However, in mice JAM-C was not detected on leukocytes but instead on fibroblasts and smooth muscle cells [160]. JAM-B expression is restricted to ECs. Endothelial and epithelial JAMs are localized at adherens junctions but primarily at tight junctions, which are intercellular adhesion complexes that delimitate the apical and basolateral plasma membrane domains and regulate intercellular communication and paracellular transport [24]. Altogether, JAMs regulate paracellular permeability, cell adhesion and polarity and participate in leukocyte transmigration. Tissue inflammation can induce the redistribution of endothelial JAMs to the luminal side and upregulate JAM expression, resulting in increased arrest of leukocytes on the endothelial layer, followed by enhanced transendothelial migration. However, effects are variable and highly dependent on the nature of the inflammatory stimuli [4,160,161,163]. In the human liver, JAM-A expression was verified [128] though its exact localization still needs to be determined, whereas JAM-B was not detectable so far with the available tools [164]. We observed JAM-C on vascular ECs and smooth muscle cells in biopsies of AIH patients that had reached complete histological remission, suggesting constitutive expression of JAM-C in these cell types. In biopsies of AIH, PBC and PSC patients, JAM-C levels were high in fibrotic areas on myofibroblasts, cholangiocytes and infiltrated leukocytes [164]. In the murine liver, we detected ubiquitous JAM-A expression, which was not upregulated upon chronic CCl_4_ treatment [152]. In contrast, chemically induced liver fibrosis was accompanied by increased JAM-B and JAM-C levels on sinusoidal ECs and de novo synthesis of JAM-C by myofibroblasts. Vascular ECs and smooth muscle cells expressed JAM-C constitutively [152]. In mouse models of PBC and of AIH, but not PSC, sinusoidal ECs showed also increased JAM-B expression, whereas JAM-C levels remained unchanged when compared to wild-type mice. Again, dependent on the disease model used, different types of murine myofibroblasts started to express JAM-C: HSCs in the models of all three immune-mediated liver diseases (AIH, PBC, and PSC), portal fibroblasts in the PSC model and capsular fibroblasts in the AIH model [164]. In myofibroblastic HSCs and portal fibroblasts, JAM-C was localized at cell–cell junctions at the terminal portion of stress fibers. Treatment of myofibroblasts with a soluble recombinant JAM-C fragment decreased JAM-C localization at cell–cell contacts and reduced cell motility, contractility, as well as expression of the differentiation marker αSMA. Furthermore, in 3D matrigel cultures, capillarized sinusoidal ECs formed longer tubes when co-cultured with myofibroblasts compared to sinusoidal ECs alone, illustrating the pericyte function of HSCs. In the same setting, soluble recombinant JAM-C fragment reduced the number of myofibroblasts associated with sinusoidal EC tubes and decreased tube length, suggesting that JAM-C-mediated myofibroblast/EC interaction supported tube formation and stability [152]. Importantly, genetic knock-out of JAM-B or treatment with soluble recombinant JAM-C fragment inhibited fibrogenesis in the AIH mouse model without affecting hepatic leukocyte infiltration [164], suggesting that blockade of sinusoidal EC/pericyte or pericyte/pericyte interactions might be a strategy to block fibrosis independent of the cause of chronic liver damage.

### 9.6. Nectins and Nectin-Like Receptors

Members of the nectin subfamily are present in cadherin-based adherens junctions but also in tight junctions and form lateral homodimers that can engage in homophilic or heterophilic adhesion with other nectins or nectin-like receptors [23,165]. Although nectins and nectin-like proteins have been detected in the liver [166] and hepatic expression of some members is upregulated upon CCl_4_ treatment or in cirrhotic tissue [167,168], the role of these IgCAMs in liver inflammation and fibrosis has not yet been analyzed in detail.

## 10. Non-Classical Adhesion Molecules

### 10.1. VAP-1

Vascular adhesion protein 1 (VAP-1) is a homodimeric transmembrane protein with amine oxidase activity, which is constitutively expressed on adipocytes and smooth muscle cells, as well as on ECs in lymph nodes, the gastrointestinal tract and the liver vasculature [29,33]. Described ligands are Siglec-9 (on granulocytes and monocytes) and Siglec-10 (on B lymphocytes, monocytes and eosinophils) (Table 2) [124]. In biopsies of patients with PBC, PSC or alcoholic liver disease, VAP-1 levels were increased on ECs and myofibroblasts but VAP-1 was absent on cholangiocytes and hepatocytes. Further, VAP-1 amine oxidase activity was higher in PSC livers than normal livers [169]. Studies with human sinusoidal ECs and animal models of liver inflammation and fibrosis demonstrated that VAP-1 controls hepatic recruitment of CD4^+^ and CD8^+^ T cells, granulocytes and monocytes [33]. Interestingly, VAP-1 amine oxidase activity had an effect on leukocyte trafficking too, such that both blockade of VAP-1 with monoclonal antibodies and with small molecule enzyme inhibitors showed distinct cell-specific inhibitory effects [170]. Further, steatohepatitis and CCl_4_-induced liver fibrosis in mice were reduced due to VAP-1-deficieny or antibody blockade, which protected from hepatic infiltration by different leukocyte subsets [171]. In these animal models, the effect of VAP-1 on hepatic immigration of leukocytes was also dependent on amine oxidase activity [171]. Amine oxidase generates chemical compounds like aldehyde, ammonia or H_2_O_2_, which can trigger NF-қB-dependent expression of CAMs in the liver, like ICAM-1, VCAM-1 or MAdCAM-1, further increasing leukocyte adhesion [124]. These results further point out that sinusoidal ECs capture leukocytes with the help of IgCAMs and VAP-1, rather than selectins. Similar to other CAMs, enzymatically cleaved VAP-1 fragments can be released from ECs, resulting in higher circulating levels of soluble VAP-1 in patients with fibrosis due to alcoholic liver disease, PBC and PSC when compared to normal controls or patients with rheumatoid arthritis or inflammatory bowel disease [33,172]. Increased serum VAP-1 levels have also been reported in patients with diabetes, obesity and the metabolic syndrome [171].

### 10.2. MAdCAM-1

Under normal conditions, mucosal addressin cell adhesion molecule 1 (MAdCAM-1), which binds to α4β1, α4β7 and L-selectin (Table 2), is expressed almost exclusively on vascular ECs in the gastrointestinal tract and on ECs of the gut-associated lymphoid tissue [124]. However, in inflammatory liver diseases, MAdCAM-1 expression is induced on vascular ECs in the portal tract and is more prominent in livers of patients with PSC and AIH than in livers of patients with PBC or HCV infection [5,124,173]. Similar to other CAMs, MAdCAM-1 levels are upregulated in the presence of TNFα and IL-1β. In addition, MAdCAM-1 hepatic expression can be induced by VAP-1, suggesting that increased VAP-1 levels can trigger MAdCAM-1 synthesis in portal ECs, which in turn allows α4β7-positive T cells that were activated in the gut to immigrate to the liver. These results may help to explain why PSC in about 70% of cases is associated with inflammatory bowel disease (IBD) [5,124]. Essentially, liver inflammation may be partly induced by typically gut-homing effector memory T cells, which by mistake have been traveling to the liver. Strikingly, in two models of steatohepatitis, MAdCAM-1-deficient mice were protected from disease and showed an increase in macrophages and immune-suppressive regulatory T cells, whereas β7-deficient mice responded with stronger disease progression and fibrosis as well as higher numbers of liver-infiltrating neutrophils compared to wild-type mice [174]. With regards to antifibrotic therapies, these data suggest that complete inhibition of β7-integrin might increase the risk of hepatic injury.

### 10.3. Stabilins

Stabilin-1, also called FEEL-1 (fasciclin, EGF-like, laminin-type EGF-like, and link domain-containing scavenger receptor 1) or CLEVER-1 (common lymphatic endothelial and vascular endothelial receptor-1) is a scavenger receptor with multifunctional properties. Its expression is inducible by proinflammatory and angiogenic factors in conventional vascular ECs and constitutive in unconventional non-continuous sinusoidal ECs in spleen, lymph nodes and liver (Table 2) [30]. Importantly, compared to normal liver, sinusoidal stabilin-1 levels are upregulated in biopsies of patients with PBC, PSC, AIH, and alcoholic liver disease [175]. In vitro studies with human hepatic sinusoidal ECs showed that stabilin-1 mediates transendothelial migration of CD4^+^FoxP3^+^ regulatory T cells, but not CD4^+^CD25^-^ or CD8^+^ effector T cells [175]. These findings suggest a protective function of stabilin-1 due to its potential to increase the number of anti-inflammatory regulatory T cells at sites of chronic inflammation. In addition, stabilin-1 played a role in B cell transendothelial migration [175]. Interestingly, naïve stabilin-1-deficient mice showed stronger basal and CCl_4_-induced hepatic collagen fiber deposition than wild-type mice, suggesting amplified fibrogenesis in the absence of stabilin-1 protective functions [176]. The authors identified a stabilin-1^+^ F4/80^+^ intrahepatic macrophage subset, which was present only in response to liver injury, both in mice and men. Such macrophages were shown to execute liver protective functions as they took up and cleared profibrogenic lipid peroxidation products generated during oxidative stress in a stabilin-1-dependent manner and concomitantly reduced the expression of the profibrogenic chemokine CCL3 [176]. These findings suggest stabilin-1 as potential therapeutic target in antifibrotic therapies. Stabilin-2 is also expressed on sinusoidal ECs in normal human and mouse livers and has been shown to bind peripheral blood lymphocytes via αMβ2 integrin. However, further studies which explore the functions of stabilin-2 in liver disease in more detail are needed [30].

## 11. Adhesion Molecules as Hepatic Fibrosis Markers and as Therapeutic Targets

Liver biopsies are the gold standard for disease monitoring, although this invasive technique has several disadvantages, like abdominal bleeding. Therefore, serological fibrosis markers, as for example analyzed by the FibroTest (markers: alanine aminotransferase, α-2-macroglobulin, haptoglobin, apolipoprotein A-1, bilirubin, γ-glutamyltranspeptidase), which allow non-invasive early diagnosis and close follow-up during therapy are needed to identify the stage of disease progression. As mentioned before, chronic hepatic injury triggers increased synthesis of many CAMs and proteolytic shedding releases peptides thereof, which can be readily detected in the serum, but they do not allow to draw conclusions on disease etiology or type of hepatic disease. For example, serum concentrations of ICAM-1 and VCAM-1 partially represent the level of hepatocellular damage as they correlate with aspartate aminotransferase activity and with fibrosis [140,177,178,179,180,181]. However, the source of circulating ICAM-1 in PBC patients are probably activated leukocytes rather than damaged cholangiocytes, since when analyzed in vitro these cholangiocytes did not release ICAM-1 [140]. Further, circulating ICAM-1 and VCAM-1 do not exclusively result from liver diseases but are detected in other malignancies as well [182,183]. A higher specificity may be offered by VAP-1, which shows a more restricted expression pattern [33,171]. Nevertheless, changes in CAM concentrations in the serum seem not adequate as liver fibrosis markers. More reliable as diagnostic applications are imaging techniques, which use CAMs to target tracers to the site of interest, as demonstrated by many preclinical studies in the atherosclerosis field, which have shown that VCAM-1, ICAM-1 or PECAM-1 can target nanoparticles to the site of inflamed vasculature [184]. For example, magnetofluorescent nanoparticles modified with a peptide homologous to the alpha chain of α4β1 (VLA-4) have been used to evaluate VCAM-1 expression via fluorescence imaging or magnetic resonance imaging. Applied to the liver field, an interesting approach is to detect capillarized sinusoidal ECs and activated HSCs, using synthetic αVβ3 ligands. If sensitive enough, such ligands may allow to detect fibrogenesis at an early stage when myofibroblast numbers have increased but the amount of generated fibrotic ECM is not yet high enough to distinctly influence tissue elasticity, a parameter which is already routinely determined by transient elastography [185]. To this end, (99m)Tc-labeled cyclic RGD pentapeptide as a radiotracer for single photon emission computed tomography and a nanoprobe labeled with cyclic RGD pentapeptide (cRGDyK) as a magnetic resonance imaging tracer have been tested successfully in CCl_4_- and thioacetamide-treated rats and mice, respectively [186,187].

Liver fibrosis is a dynamic and bidirectional process, such that removal of the causative trigger, e.g., with antiviral therapy, to eliminate hepatitis B or C virus or avoidance of liver-toxins, such as alcohol, can stop fibrosis progression and lead to substantial fibrosis reversion [188,189]. However, in most cases, the etiology of the liver disease is unknown, and its removal or obviation are impossible. Therefore, antifibrotic agents are urgently needed, and they should preferentially act exclusively in the fibrotic milieu in order to prevent systemic side-effects [190,191,192,193,194]. In this regard, nanoparticles are an attractive tool, since they naturally accumulate predominantly in the liver [195,196]. Antifibrotic biopharmaceuticals, such as recombinant monoclonal antibodies, could target CAMs following three strategies: (A) CAMs which support homing of proinflammatory/profibrotic cell types will be blocked; (B) CAMs which show intrinsic profibrotic activity will be directly inhibited; or (C) CAMs will be used to target antifibrotic drugs to a specific cell type. Examples of strategy A are antibodies to α4β7 or MAdCAM-1, which inhibit homing of gut-primed effector T cells to the gastrointestinal tract via endothelial MAdCAM-1 or VCAM-1 and therefore are effective in patients with IBD. Such antibodies could as well be beneficial for PSC patients with associated IBD to block hepatic immigration of gut-primed memory T cells. Unfortunately, the conducted clinical studies with anti-α4β7 antibodies have generated conflicting results and anti-MAdCAM-1 antibodies have not been tested in PSC patients so far [124]. An anti-VAP-1 antibody was tested in PSC patients, but results are not yet available [197]. Of note, blocking VAP-1 reduced hepatic leukocyte recruitment in four different animal models of liver injury [124]. For strategy B, integrins αVβ1 and αVβ6 are suitable, since they show a certain cell type-specific expression (mostly on myofibroblasts and hepatic ECs, respectively) and they release the profibrotic factor TGFβ. In fact, a small molecule αVβ1 inhibitor, which was able to down-regulate TGFβ-induced signaling in vivo, showed a significant therapeutic effect in reducing CCl_4_-induced hepatic fibrosis in mice [98]. In three biliary fibrosis models in rodents, blockade of αVβ6 by the nonpeptide antagonist EMD527040 or by a monoclonal anti-αVβ6 antibody reduced fibrosis progression, cholangiocyte proliferation and binding to fibronectin and the release of active TGFβ without inducing cholangiocyte apoptosis or affecting other cell types [108,109]. Further, therapeutic administration of the αV integrin-specific-RGD peptidomimetic antagonist CWHM 12 also reduced pre-existing hepatic fibrosis, most probably again due to lowered TGFβ1 activation [97]. In addition, blocking integrin-linked signaling pathways may work as antifibrotic therapy: Ursodeoxycholyl lysophosphatidylethanolamide (UDCA-LPE), a synthetic bile acid–phospholipid conjugate, acts as a heterobivalent ligand for integrins and lysophospholipid receptor-1 and as such was able to induce lipid raft-mediated internalization of β1-integrin and subsequent inhibition of fibrogenic β1 signaling in vitro [198]. Bansal et al. have demonstrated a link between α11-integrin and the hedgehog signaling pathway [86]. Inhibition of hedgehog by LDE225 has reduced fibrotic parameters and α11 expression in CCl_4_-treated mice and in human liver slices ex vivo [86]. Further, pharmacological inhibition of P21-activated kinase (PAK) or the mechanosensitive factor Yes-associated protein-1 (YAP-1), two mediators of β1-integrin-controlled profibrotic signaling, reduced liver fibrosis induced by CCl_4_ and bile duct ligation [85]. Yet another antifibrotic approach may be the inhibition of sinusoidal EC/pericyte or pericyte/pericyte interactions since soluble recombinant JAM-C fragment prevented fibrogenesis in an AIH mouse model without affecting hepatic leukocyte infiltration [164]. However, one has to bear in mind that in men infiltrating T lymphocytes, NK cells or dendritic cells also express JAM-C. Therefore, JAM-C blocking reagents need to be targeted specifically to pericytes to avoid interference with leukocyte migration. To target antifibrotic agents to the liver (strategy C), drug-loaded nanoparticles were modified with RGD peptide to allow binding to integrins upregulated on activated HSCs. Examples of successfully delivered compounds are IFNα1b and oxymatrine, a herbal compound, which induces apoptosis in activated HSCs, as both were efficient in blocking liver fibrosis induced by bile-duct ligation or CCl_4_ in rodents [199,200].

Although focused targeting on specific CAMs may indeed prove beneficial for the patient, such therapeutic approaches are usually tailored to a distinct type of human disease or to a specific animal model. However, etiology and course of chronic liver disease in men are highly complex. As outlined in the previous chapters, many CAMs promote inflammation and fibrogenesis since they support homing of proinflammatory cells to the liver. Depending on the insult, these cells can make use of different types of CAMs, e.g., neutrophils use CD44 and hyaluronan during infection but β2-integrins during sterile injury [201]. Furthermore, certain CAMs, like PECAM-1 or stabilin-1 can also fulfill protective functions, e.g., by mediating the recruitment of anti-inflammatory cells to the site of hepatic inflammation [175]. The role of a specific CAM in fibrogenesis depends on the type of hepatic damage, as this event defines the liver-immigrating active immune cell repertoire by the release of specific chemokines. Of relevance are different T cell subsets since they define the nature and outcome of hepatic inflammation. For example, chronic hepatitis C virus infection and PBC are both dominated by a Th1 immune response, whereas in PSC a Th2 response may be involved and in alcoholic and non-alcoholic steatohepatitis Th17 cells and neutrophils are of importance [6]. However, since in most situations overlapping mechanisms are active, an effective antifibrotic therapy may need to target additional players besides CAMs.

## 12. Conclusions

**Figure 1:**
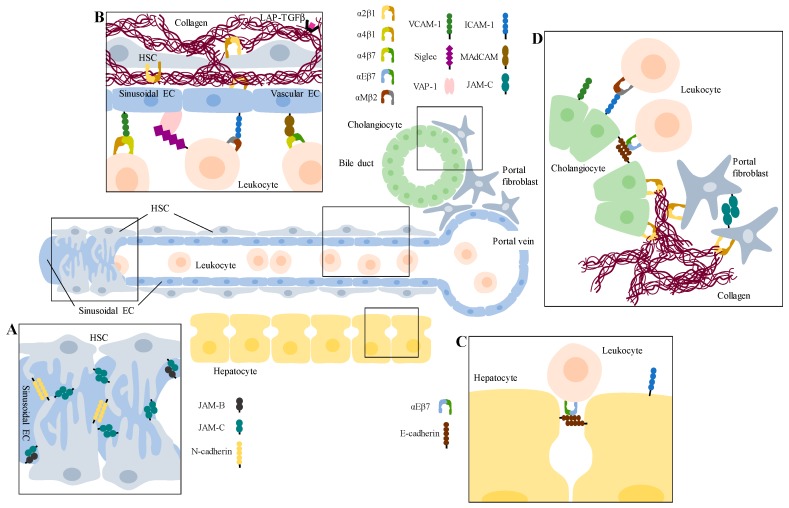
Overview of hepatic cell interactions mediated by CAMs during liver inflammation and fibrosis. Displayed is a sinusoidal channel near a portal vein and a bile duct. (**A**) The microvessel is covered by activated hepatic stellate cells (HSCs), which act as mural cells. They interact with each other via N-cadherin and JAM-C homophilic binding and with sinusoidal endothelial cells (ECs) via JAM-B/JAM-C interaction. (**B**) HSCs are the main producers of fibrotic extracellular matrix (ECM), like collagen I. Tethered to the ECM is LAP-TGFβ. Chemokine-attracted leukocytes get recruited to capillarized sinusoidal ECs by binding via integrins to members of the immunoglobulin (Ig) superfamily of cell adhesion molecules (IgCAMs) or to non-classical CAMs like VAP-1, or they bind to MAdCAM-1 on vascular ECs before they transmigrate the endothelial wall and interact later on with (**C**) hepatocytes. (**D**) In biliary disease, portal fibroblasts also get activated, secrete fibrotic ECM and bind to each other via JAM-C. Further, leukocytes attach to cholangiocytes, e.g., via integrin/IgCAM binding.

Cell–ECM binding and cell–cell adhesion are dependent on CAMs, which act as adhesive structures and as signal transducers. They control a multitude of processes both in liver homeostasis and disease. As outlined in this review and summarized in Table 3 and Figure 1, CAMs participate in multifaceted interactions, which can either support fibrogenesis very broadly by releasing profibrogenic TGFβ or mediating leukocyte immigration or rather cell-type specifically by reinforcing pericyte mural functions. Several candidate CAMs have been evaluated as targets to prevent and/or reverse hepatic inflammation and fibrosis. Although evidence from animal models as well as from some initial clinical trials are promising, much more progress has still to be made in order to provide a successful therapy for patients with liver fibrosis. Thus, in order to find novel therapeutic interventions for inflammation-induced liver fibrosis, it will be important to further identify critical profibrotic factors and to investigate the interplay between CAMs present on liver-resident as well as invading cells.

## Figures and Tables

**Table 1:** Members of the selectin and integrin group of CAMs and their ligands/counter-receptors expressed in the healthy and inflamed liver.

Adhesion Molecule	Adhesion Molecule Expressing Resident and Immigrated Liver Cell Type	ECM Ligand and Counter-Receptor	Counter-receptor Expressing Resident and Immigrated Liver Cell Type
**Selectins** E-selectin P-selectin L-selectin	**vEC** **vEC**, P T	PSGL-1 PSGL-1 **MECA-79**, MAdCAM-1	LC LC EC
**Integrins** α1β1 (VLA-1) α2β1 (VLA-2) α3β1 (VLA-3) α4β1 (VLA-4) α5β1 (VLA-5) α6β1 (VLA-6) α11β1 αLβ2 (LFA-1) αMβ2 (Mac-1) αXβ2 (p150,95) αDβ2 αVβ1 αVβ3 αVβ5 αVβ6 αVβ8 α4β7 αEβ7	sEC, vEC, H, HSC C, sEC, vEC, periportal **H**, HSC C, vEC, **H** **sEC**, LC C, sEC, **vEC**, **H**, **HSC** C, vEC, **H** HSC LC LC LC LC HSC EC, HSC EC, HSC **C**, **H** H, HSC T T, D	CL, LN CL, LN LN FN, JAM-B, MAdCAM-1, VCAM-1 FN LN CL ICAMs, JAM-A ICAM, JAM-C ICAM, JAM-C ICAM, VCAM FN, LAP-TGFβ FN, TN, VN, LAP-TGFβ, JAM-A, JAM-C VN, LAP-TGFβ FN, TN, LAP-TGFβ VN, LAP-TGFβ FN, MAdCAM-1, VCAM-1 E-cadherin	C, EC, H, HSC EC, **EpC**, HSC, LC EC, EpC, HSC EC, C, H, HSC EC, C, H, HSC EC, C, H, HSC EC C, H, HSC

Mentioned are those selectins and integrins which have been analyzed in connection with liver inflammation and fibrosis in rodents and men. Liver cells or liver-infiltrating leukocytes expressing these CAMs and the corresponding counter-receptors are listed. Cell types in bold show expression only under inflammatory conditions. Abbreviations: C, cholangiocyte; sEC, sinusoidal endothelial cell; CL, collagen; D, dendritic cell; vEC, vascular endothelial cell; EpC, epithelial cell; FN, fibronectin; H, hepatocyte; HSC, hepatic stellate cell; ICAM, intercellular adhesion molecule; JAM, junctional adhesion molecule; LAP, latency-associated peptide; LC, leukocyte; MAdCAM, mucosal addressin cell adhesion molecule; P, platelet; PECAM, platelet-endothelial cell adhesion molecule; PSGL-1, P-selectin glycoprotein ligand-1; T, T cell; TGFβ, transforming growth factor beta; TN, tenascin-C; VCAM, vascular cell adhesion molecule; VN, vitronectin.

**Table 2:** Members of the cadherin group, the immunoglobulin superfamily of CAMs and some non-classical CAMs and their counter-receptors expressed in the healthy and inflamed liver.

Adhesion Molecule	Adhesion Molecule Expressing Resident and Immigrated Liver Cell Type	Counter-Receptor	Counter-Receptor Expressing Resident and Immigrated Liver Cell Type
**Cadherins**			
Desmoglein Desmocollin VE-cadherin E-cadherin N-cadherin Cadherin-11	EpC EpC EC C, periportal H, HSC H, **HSC** H, HSC, M	Desmoglein, Desmocollin Desmocollin, Desmoglein VE-cadherin E-cadherin, [αEβ7, KLRG1] N-cadherin Cadherin-11	EpC EpC EC C, periportal H, HSC, [T, D, NK] H, **HSC** H, HSC, M
**IgCAMs**			
ICAM-1 VCAM-1 PECAM-1 NCAM JAM-A JAM-B JAM-C	C, EC, **H**, **HSC** C, EC, **HSC** EC, LC **C**, **HSC**, **PF** EC, EpC, LC, P mouse sEC and vEC **C**, sEC, vEC, **HSC**, **PF**, SM human LC	ICAM-1, β2 integrins like αLβ2 VCAM-1, α4β1 PECAM-1, [αVβ3] NCAM JAM-A, αLβ2, αVβ3 JAM-C, JAM-B, [α4β1] JAM-B, JAM-C, [αMβ2, αXβ2, αVβ2]	EC, **C**, **H**, **HSC**, LC EC, **C**, **HSC**, LC EC, [T] **C**, **HSC**, **PF** EC, EpC, P, LC mouse sEC and vEC, [LC] C, sEC, vEC, **HSC**, **PF**, SM, [LC]
**Non-classical**			
VAP-1 MAdCAM-1 Stabilin-1	vEC, sEC, **HSC** **vEC** sEC, vEC	Siglec-9, Siglec-10 α4β1, α4β7, L-selectin αLβ2	LC T Treg, B

Mentioned are those cadherins, IgCAMs and non-classical CAMs which have been analyzed in connection with liver inflammation and fibrosis in rodents and men. Liver cells or liver-infiltrating leukocytes expressing these CAMs and the corresponding counter-receptors are listed. Cell types in bold show expression only under inflammatory conditions. Abbreviations: B, B cell; C, cholangiocyte; D, dendritic cell; sEC, sinusoidal endothelial cell; vEC, vascular endothelial cell; EpC, epithelial cell; H, hepatocyte; HSC, hepatic stellate cell; ICAM, intercellular adhesion molecule; JAM, junctional adhesion molecule; KLRG1, killer cell lectin-like receptor G1; LC, leukocyte; M, macrophage; MAdCAM, mucosal addressin cell adhesion molecule; NCAM, neuronal cell adhesion molecule; NK, natural killer cell; P, platelet; PECAM, platelet-endothelial cell adhesion molecule; PF, portal fibroblast; SM, smooth muscle cell; T, T cell; Treg, regulatory T cell; VAP-1, vascular adhesion protein; VCAM, vascular cell adhesion molecule.

**Table 3:** CAM mediated hepatic cell interactions and their impacts on cell behavior during liver fibrogenesis.

Liver Cell	Interaction Type	Functions/Effects	CAM Group Involved
HSC	HSC/ECM	Induction of TGFβ release Coordination of fibrogenic activation and perpetuation Perception of ECM composition Support of fibrotic ECM synthesis Coordination of motility and contractility	Integrins
	HSC/HSC	Contribution to fibrogenic activation and survival Coordination of contractility and motility	Cadherins, IgCAMs
	HSC/sEC	Vessel wall stabilization and diameter control Support of fibrosis-associated neovascularization	IgCAMs
	HSC/T cell	HSC activation after phagocytosis of T cells	IgCAM (ICAM-1)
sEC	sEC/ECM	Coordination of capillarization, proliferation and motility Support of fibrosis-associated neovascularization	Integrins
	sEC/sEC	Contribution to tissue integrity, cell polarity, functionality Coordination of motility and neovascularization	Cadherins, IgCAMs
	sEC/HSC	sECs influence HSCs rather by soluble factors than direct cell-cell contact	IgCAMs
	sEC/leukocyte	Leukocyte recruitment	Integrins, cadherins, IgCAMs, non-classical CAMs
EpC	EpC/ECM	Coordination of polarity, homeostasis, proliferation Coordination of motility	Integrins
	EpC/EpC	Contribution to tissue integrity, cell polarity, proliferation and functionality Coordination of motility	Cadherins, IgCAMs
	EpC/leukocyte	Leukocyte recruitment, support of leukocyte survival	Integrins, cadherins, IgCAMs

Listed are those CAM-controlled interactions of liver-resident cells and immigrated leukocytes which support hepatic fibrogenesis. Abbreviations: EpC, epithelial cell; sEC, sinusoidal endothelial cell; HSC, hepatic stellate cell; IgCAMs, immunoglobulin (Ig) superfamily of CAMs; TGFβ, transforming growth factor beta.
